# Identification of the Main Regulator Responsible for Synthesis of the Typical Yellow Pigment Produced by Trichoderma reesei

**DOI:** 10.1128/AEM.01408-16

**Published:** 2016-09-30

**Authors:** Christian Derntl, Alice Rassinger, Ewald Srebotnik, Robert L. Mach, Astrid R. Mach-Aigner

**Affiliations:** a Research Area Biochemical Technology, Institute of Chemical Engineering, TU Wien, Vienna, Austria; b Bioresources, Plant and Food Science, Institute of Chemical Engineering, TU Wien, Vienna, Austria; HKI and University of Jena

## Abstract

The industrially used ascomycete Trichoderma reesei secretes a typical yellow pigment during cultivation, while other Trichoderma species do not. A comparative genomic analysis suggested that a putative secondary metabolism cluster, containing two polyketide-synthase encoding genes, is responsible for the yellow pigment synthesis. This cluster is conserved in a set of rather distantly related fungi, including Acremonium chrysogenum and Penicillium chrysogenum. In an attempt to silence the cluster in T. reesei, two genes of the cluster encoding transcription factors were individually deleted. For a complete genetic proof-of-function, the genes were reinserted into the genomes of the respective deletion strains. The deletion of the first transcription factor (termed yellow pigment regulator 1 [Ypr1]) resulted in the full abolishment of the yellow pigment formation and the expression of most genes of this cluster. A comparative high-pressure liquid chromatography (HPLC) analysis of supernatants of the *ypr1* deletion and its parent strain suggested the presence of several yellow compounds in T. reesei that are all derived from the same cluster. A subsequent gas chromatography/mass spectrometry analysis strongly indicated the presence of sorbicillin in the major HPLC peak. The presence of the second transcription factor, termed yellow pigment regulator 2 (Ypr2), reduces the yellow pigment formation and the expression of most cluster genes, including the gene encoding the activator Ypr1.

**IMPORTANCE** Trichoderma reesei is used for industry-scale production of carbohydrate-active enzymes. During growth, it secretes a typical yellow pigment. This is not favorable for industrial enzyme production because it makes the downstream process more complicated and thus increases operating costs. In this study, we demonstrate which regulators influence the synthesis of the yellow pigment. Based on these data, we also provide indication as to which genes are under the control of these regulators and are finally responsible for the biosynthesis of the yellow pigment. These genes are organized in a cluster that is also found in other industrially relevant fungi, such as the two antibiotic producers Penicillium chrysogenum and Acremonium chrysogenum. The targeted manipulation of a secondary metabolism cluster is an important option for any biotechnologically applied microorganism.

## INTRODUCTION

**FIG 1:**
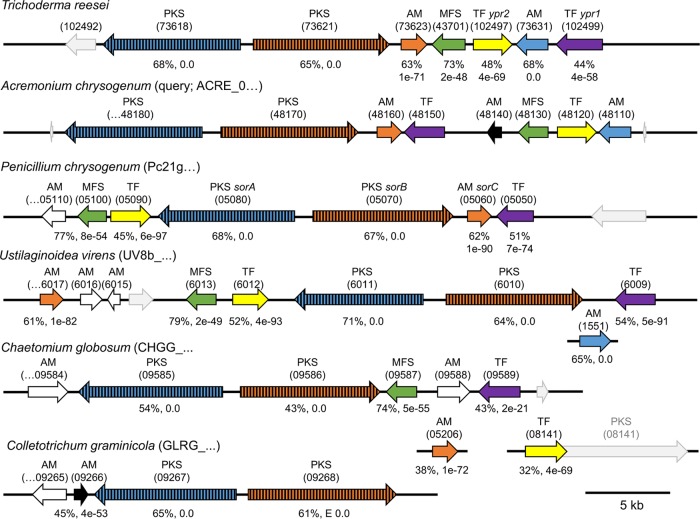
Schematic presentation of the secondary metabolism gene cluster conserved in seven ascomycetes. Arrows with black frames indicate genes belonging to the secondary metabolism gene clusters. Arrows with the same color and pattern represent homologs. White arrows represent (putative) genes that do not have homologs in the clusters of the other organisms. Gray arrows with gray frames indicate genes in proximity to the cluster genes. Orientation, distance, and size are set to scale and comparable. The black bar at the bottom represents 5 kb. If genes are depicted on an additional line, they are located on different scaffolds. The protein sequences of A. chrysogenum were used as query for a DELTA-BLAST analysis (27); the similarities of the homologs are represented by the percent identity and E value below the arrows. PKS, polyketide synthase; AM, auxiliary modifier; MFS, transporter of the multifacilitator superfamily; TF, transcription factor. Protein IDs are given in parentheses (the beginning of the ID after the species name and the variable part above the respective arrow). The cluster of Colletotrichum sublineola is not presented because it is essentially the same as the one of Colletotrichum graminicola.

The ascomycete Trichoderma reesei (teleomorph, Hypocrea jecorina [1]) is routinely used for the industrial production of cellulases, hemicellulases, and recombinant proteins and thus is an object of intense (academic) research (2). In contrast to its close relatives, T. atroviride and T. virens, T. reesei secretes a typical yellow pigment during growth (3, 4). This yellow pigment needs to be removed during the downstream process of industry-scale protein production. Obviously, it is of interest to identify the genes that are responsible for the synthesis of the yellow pigment. Since pigments in fungi are often secondary metabolites (5, 6) and T. reesei possesses, in contrast to other species such as T. atroviride and T. virens (7), only a small set of secondary metabolism-related genes, we decided to perform a comparative genomic analysis. In the genome of T. reesei 11 polyketide synthase (PKS)-encoding genes are annotated (8), of which only two are neither present in T. atroviride nor present in T. virens (9). Interestingly, these two are arranged tail to tail in a cluster that is conserved in a set of rather distantly related ascomycetes (Fig. 1). In fact, clusters containing homologs of both PKS in the direct vicinity are only found in the genomes of Acremonium chrysogenum (10), Penicillium chrysogenum (11), Ustilaginoidea virens (GenBank JHTR00000000.1), Chaetomium globosum (12), Colletotrichum graminicola (13), and Colletotrichum sublineola (14). Figure 1 gives a schematic representation of the clusters in these species. In nearly all organisms the two PKS-encoding genes are accompanied by genes encoding a transporter, one or two transcription factors, and further auxiliary modifiers (AMs), e.g., reductases, monooxygenases, and dehydrogenases. In the two Colletotrichum species, the clusters do not contain a transporter or a transcription factor. This, together with the fact that the cluster is embedded in an AT-rich region in Colletotrichum graminicola, suggests a nonfunctionality in Colletotrichum. In Colletotrichum sublineola the scaffold breaks off at the edges of the cluster. However, in P. chrysogenum, genomic mutational analysis provided strong indications that this cluster is responsible for the production of a yellow pigment (15). Just recently, Salo et al. demonstrated that the yellow pigment is a sorbicillinoid originating from the cluster (16). In T. reesei the chemical nature of the pigment was not described. We decided to investigate the function of the cluster in T. reesei by a reverse genetic approach. Since secondary metabolism gene clusters can be activated by inherent transcription factors (17, 18), we assumed that the deletion of the two transcription factors in the cluster might result in silencing of the whole cluster.

Consequently, during this study, we deleted the transcription factors in the T. reesei secondary metabolism cluster (TF 102497 and TF 102499) and elucidated their role regarding the formation of the yellow pigment. We could link the entire formation of yellow pigment(s) to the mentioned gene cluster by performing photometrical analysis, high-pressure liquid chromatography (HPLC) analysis, and quantitative PCR (qPCR) analysis. In addition, we could identify one compound originating from this cluster as sorbicillin. However, the focus of the present study was to determine the regulatory roles of the two transcription factors in the cluster. We could demonstrate that the deletion of one of the two transcription factors shuts off the whole cluster and that an internal feedback loop seems to be present. Our results stress the significance of this gene cluster in the case of T. reesei and all other ascomycetes bearing it, regardless of whether the formation of the resulting compounds is anticipated or should be avoided.

## MATERIALS AND METHODS

### Strains and cultivation conditions.

T. reesei strains QM6aΔ*tmus53* (QM6a) (19), QM6aΔ*tmus53*Δ*pyr4* (Δ*pyr4*) (20), QM6aΔ*tmus53*Δ*ypr1* (Δ*ypr1*, this study), QM6aΔ*tmus53*Δ*ypr1*Re*ypr1* (Re*pyr1*; this study), QM6aΔ*tmus53*Δ*ypr2* (Δ*ypr2*; this study), and QM6aΔ*tmus53*Δ*ypr2*Re*ypr2* (Re*ypr2*; this study) were maintained on malt extract (MEX) agar at 30°C. If applicable, uridine and hygromycin B were added to final concentrations of 5 mM and 113 U/ml, respectively. Mandels-Andreotti (MA) medium (21) without peptone containing 1% d-glucose was used as a minimal medium.

For determination of growth on plates, T. reesei was pregrown on solid MA medium containing 1% d-glucose at 30°C. Equal pieces taken from overgrown agar plates were used as inoculum and plates were incubated at 30°C in darkness. Pictures were taken from the bottom of the plates. For liquid cultures, T. reesei was grown in MA medium containing 1% d-glucose at 30°C either in a volume of 250 ml in 1,000-ml Erlenmeyer flasks on a rotary shaker at 180 rpm or stationary in a volume of 1.5 ml in 12-well plates. Mycelia and supernatants were separated by filtration through Miracloth (EMD Millipore [part of Merck KGaA], Darmstadt, Germany). Mycelia were stored in liquid nitrogen.

Escherichia coli strain Top10 (Invitrogen [part of Thermo Fisher Scientific, Inc.], Waltham, MA) was used for all cloning purposes throughout this study and maintained on Luria-Bertani medium at 37°C. If applicable, ampicillin was added to a final concentration of 100 mg/ml.

### Plasmid constructions.

PCRs for cloning purposes were performed with KAPA HiFi DNA polymerase (Kapa Biosystems, Wilmington, MA) according to the manufacturer's instructions. All of the primers used are listed in Table 1. PCR products were cloned into EcoRV-digested pJET1.2 (Thermo Scientific [part of Thermo Fisher Scientific, Inc.]) and after verification of the PCR products by sequencing (Microsynth, Balgach, Switzerland), they were released for subsequent cloning purposes by digestion with suitable restriction endonucleases.

**TABLE 1:**
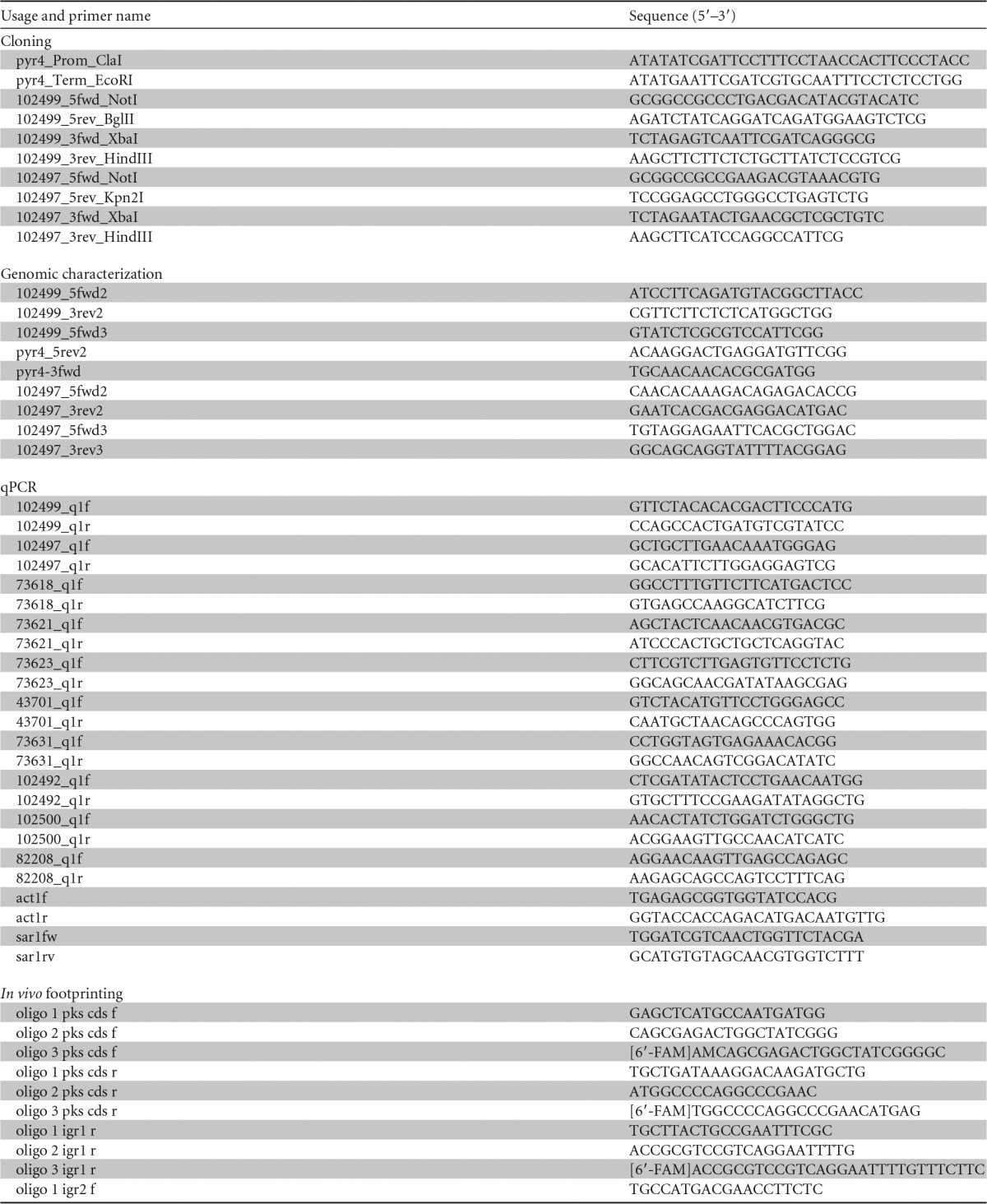

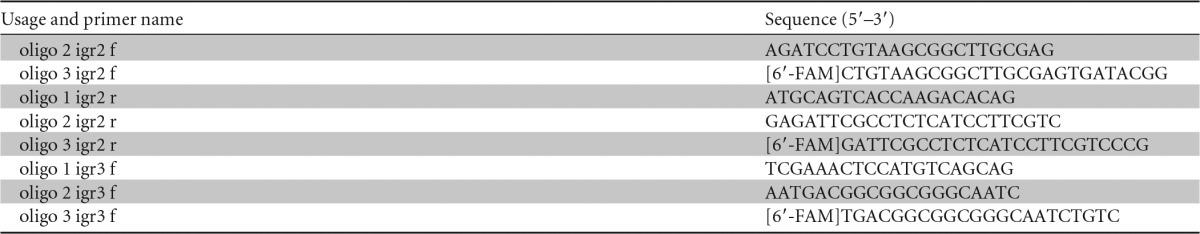
Oligonucleotides used in this study

For the construction of pJET-pyr4, the *pyr4* gene, including its own promoter and terminator, was amplified by PCR with the primers pyr4_Prom_ClaI and pyr4_Term_EcoRI using chromosomal DNA of T. reesei QM6a as a template and inserted into EcoRV-digested pJET1.2 in the same orientation as the *eco47IR* gene.

For the construction of pCD-Δypr1, the 5′- and 3′-flanks of *ypr1* were amplified by PCR with the primers 102499_5fwd_NotI and 102499_5rev_BglII or the primers 102499_3fwd_XbaI and 102499_3rev_HindIII, respectively, using chromosomal DNA of T. reesei QM6a as the template. The 3′-flank was inserted into pJET-pyr4 via restriction with XbaI and HindIII, and then the 5′-flank was added using NotI and BglII.

For the construction of pJET-ReYpr1, the *ypr1* gene, including its own promoter and terminator, was amplified by PCR with the primers 102499_5fwd3 and 102499_3rev2 using chromosomal DNA of T. reesei QM6a as the template and inserted into EcoRV-digested pJET1.2.

For the construction of pCD-Δypr2, the 5′- and 3′-flanks of *ypr2* were amplified by PCR with the primers 102497_5fwd_NotI and 102497_5rev_Kpn2I or the primers 102497_3fwd_XbaI and 102497_3rev_HindIII, respectively, using the chromosomal DNA of T. reesei QM6a as the template. The 3′-flank was inserted into pJET-pyr4 via restriction with XbaI and HindIII, and then the 5′-flank was added using NotI and Kpn2I.

For the construction of pJET-ReYpr2, the *ypr2* gene, including its own promoter and terminator, was amplified by PCR with the primers 102497_5fwd3 and 102497_3rev3 using chromosomal DNA of T. reesei QM6a as the template and inserted into EcoRV-digested pJET1.2.

### Fungal transformation.

The protoplast transformation of T. reesei was performed as described by Gruber et al. (22). Typically, 30 μg of digested plasmid DNA or 5 μg of undigested plasmid DNA (in 15 μl of sterile double-distilled H_2_O [ddH_2_O]) was used for the transformation of 10^7^ protoplasts. For selection for prototrophy, 100 μl to 2 ml of the transformation reaction was added to 20 ml of melted, 50°C warm minimal medium agar containing 1.2 M sorbitol. This mixture was poured into sterile petri dishes. For selection for resistance against hygromycin B, 100 μl to 2 ml of the transformation reaction mixture was added to 10 ml of melted, 50°C warm MEX agar containing 1.2 M sorbitol. This mixture was poured into sterile petri dishes. Subsequently, 10 ml of melted, 50°C warm MEX agar containing 1.2 M sorbitol and a double concentration of hygromycin B was poured on top of the protoplast-containing layer. The plates were incubated at 30°C for 3 to 5 days until colonies were visible. Candidates were subjected to at least three rounds of homokaryon selection by spore streakouts on selection medium plates until stable, homokaryotic strains were obtained.

### Isolation of chromosomal DNA and PCR screening.

Chromosomal DNA was isolated from mycelium by grinding in liquid nitrogen, followed by a phenol-chloroform extraction (22). RNA was degraded using RNase A (Thermo Scientific). DNA was precipitated with isopropanol, washed with 70% ethanol, and dissolved in double-distilled water (ddH_2_O). To test the genotype, 10 ng of chromosomal DNA was used as the template in a 25-μl PCR with GoTaq G2 polymerase (Promega, Madison, WI) according to the manufacturer's instructions. All of the primers used are listed in Table 1. For subsequent agarose gel electrophoresis of the DNA fragments, a GeneRuler 1-kb DNA ladder (Thermo Scientific) was applied to estimate the fragment size.

### Southern blot analysis.

Portions (15 μg) of chromosomal DNA were digested with 30 U of the indicated restriction enzymes. The resulting DNA fragments were separated by electrophoresis on an 0.8% agarose gel and then denatured in 0.4 M NaOH and transferred by capillary forces onto a Biodyne B 0.45-μm-pore-size nylon membrane (Pall Corporation, Port Washington, NY) using 10× SSC (1× SSC is 0.15 M NaCl plus 0.015 M sodium citrate). Then, 1.5 μg of biotinylated DNA probe was used for hybridization at 65°C overnight. Labeling of the probe was performed by using a Klenow fragment (exo-) (Thermo Scientific), random hexamer primers, and biotin-11-dUTP (Jena Bioscience, Jena, Germany). Signals were visualized by using Poly-HRP conjugated to streptavidin and ECL Plus Western blotting substrate (both from Thermo Scientific) on a ChemiDoc MP imaging system (Bio-Rad Laboratories, Hercules, CA).

### HPLC.

A 100-ml culture supernatant was extracted twice with 50 ml of ethyl acetate. The pooled organic phases were dried over calcium chloride, evaporated to dryness under reduced pressure, and redissolved in 2 ml of ethyl acetate. Next, 0.1 ml of the extract was evaporated under a stream of nitrogen, redissolved in 0.5 ml of acetonitrile, and filtered through a 0.45-mm-pore-size nylon membrane. Portions (10 μl) were then subjected to high-performance liquid chromatography (HPLC; Agilent 1200 system with diode array detector) on a reversed-phase column (Agilent SB-C18; 250 by 4.6 mm) at 30°C and a flow rate of 1.0 ml/min. Metabolites were eluted with acetonitrile-water-H_3_PO_4_ (100:900:1) for 1 min, followed a linear gradient adjusted to 1,000:0:1 (acetonitrile-water-H_3_PO_4_) between 1 and 40 min. The eluate was monitored at 400 nm, and complete UV-Vis spectra were determined at a 2-nm resolution.

Preparative HPLC was performed by multiple injections of metabolites in acetonitrile under conditions similar to those described above but with trifluoroacetic acid instead of H_3_PO_4_ and collecting the eluate with a fraction collector. Fractions corresponding to major peaks were pooled, freeze-dried overnight, and then derivatized by adding 80 μl of BSTFA [*N*,*O*-bis(trimethylsilyl)trifluoroacetamide], 20 μl of TMCS (trimethylchlorosilane), and 20 μl of pyridine for 45 min at 75°C. Then, 1 μl of each silylated sample was analyzed by splitless injection at 280°C into a gas chromatography-mass spectrometry (GC/MS) apparatus (Agilent Technologies, USA, model no. 7890A/5975C) equipped with a HP-5MS column (30 m by 0.25 mm [inner diameter] by 0.25 μm; Agilent Technologies) and He as the carrier gas at 30 cm/min. The oven temperature was initially held for 1 min at 100°C and then ramped from 100 to 325°C at 10°C/min, followed by a hold at 325°C for 15 min. Mass spectra were taken in scan mode over an *m/z* range of 35 to 750.

### Transcript analysis by reverse transcription-quantitative PCR (RT-qPCR).

Approximately 20 mg of harvested mycelia was homogenized in 1 ml of peqGOLD TriFast DNA/RNA/protein purification system reagent (Peqlab Biotechnologie, Erlangen, Germany) using a FastPrep FP120 BIO101 ThermoSavant cell disrupter (Qbiogene, Carlsbad, CA). RNA was isolated according to the manufacturer's instructions, and the concentration was measured using a NanoDrop 1000 spectrophotometer (Thermo Scientific). Synthesis of cDNA from mRNA was carried out using the RevertAid H Minus first-strand cDNA synthesis kit (Thermo Scientific) according to the manufacturer's instructions.

Quantitative PCRs were performed in a Mastercycler ep realplex 2.2 system (Eppendorf, Hamburg, Germany). All reactions were performed in triplicates. The amplification mixture (final volume, 25 μl) contained 12.5 μl of 2× iQ SYBR green mix (Bio-Rad), 100 nM concentrations of the forward and reverse primers, and 2.5 μl of cDNA (diluted 1:100) as the template. All of the primers used are listed in Table 1. Cycling conditions and control reactions were as described previously (23). Calculations using *sar1* and *act1* as reference genes were performed as published previously (23).

### *In vivo* footprinting analysis.

*In vivo* methylation using dimethyl sulfate, followed by ligation-mediated PCR, was performed as described previously (24). 6′-Carboxyfluorescein (6′-FAM)-labeled fragments were generated by PCR using primers (Table 1) to cover the whole intergenic region between the two PKS-encoding genes of the cluster. The analysis of fragments was performed by capillary gel electrophoresis (Microsynth), and the results were analyzed using ivFAST (24). For the previously described landscape visualization (25), the data sets of the coding and noncoding strand were combined; then, for each base pair, the mean of the output value of this base pair and of the four adjacent base pairs was calculated. The obtained mean values were converted to a logarithmic scale and expressed as the protein-DNA interaction index (PDI).

## RESULTS

### TF 102499 is essential for the production of the yellow pigment(s) in T. reesei.

**FIG 2:**
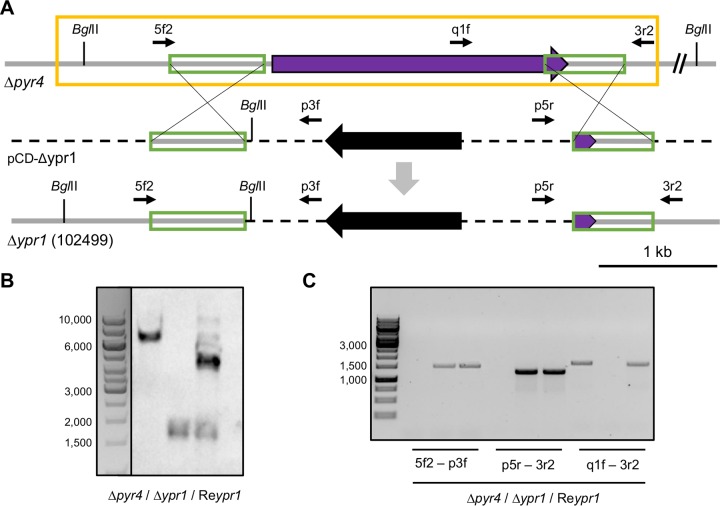
Genotypic characterization of the T. reesei TF 102499 (Ypr1) deletion and complementation strains. (A) The uridine auxotrophic strain (Δ*pyr4*) was transformed with the plasmid pCD-Δypr1, which bears the deletion cassette, in order to replace the gene encoding the TF 102499 (purple arrow) with the marker gene *pyr4* (black arrow) via homologous recombination yielding a prototrophic, TF 102499 deletion strain (Δ*ypr1*). The green frames represent 5′- and 3′-flanks for the homologous recombination. The gray arrow indicates the recombination event. Recognition sites of the endonuclease BglII and positions of the primers (thin black arrows) used for PCR analysis are depicted. 5f2, 102499_5fwd2; q1f, 102499_q1f; 3r2, 102499_3r2; p3f, pyr4-3fwd; p5r, pyr4-5rev2. Gray bars indicate fungal genomic DNA, and dashed and black bars indicate plasmid DNA. The yellow frame indicates the expression cassette used for the TF 102499 complementation. The complementation was performed via ectopic insertion, leaving the original locus deleted. (B) Southern blot using BglII-digested chromosomal DNA of the parent strain (Δ*pyr4*), the TF 102499 deletion strain (Δ*ypr1*), and the complemented strain (Re*ypr1*), as well as the 5′-flank as the probe. (C) Agarose gel electrophoresis of the fragments obtained by PCR with the indicated primers using chromosomal DNA of the parent strain (Δ*pyr4*), the TF 102499 deletion strain (Δ*ypr1*), and the complemented strain (Re*ypr1*) as templates.

**FIG 3:**
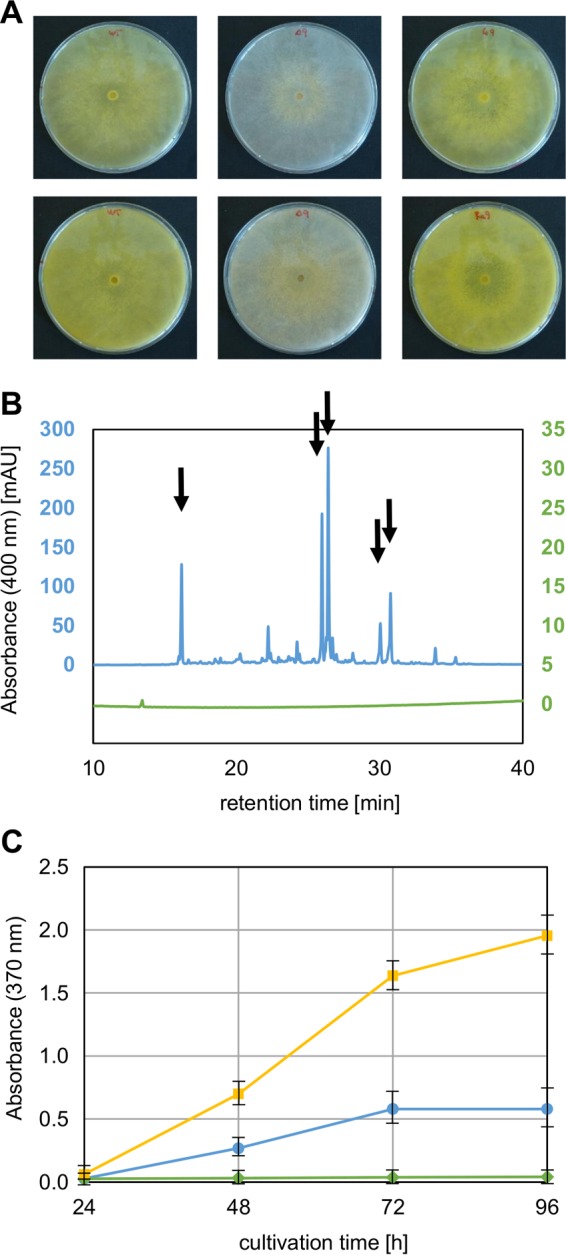
Phenotypic characterization of the T. reesei TF 102499 (Ypr1) deletion and complementation strains. (A) Pictures of the T. reesei QM6a (left), Δ*ypr1* (middle), and Re*ypr1* (right) strains grown on plates containing d-glucose for 48 h (top) and 72 h (bottom). (B) Chromatogram of a reversed-phase HPLC of ethyl acetate extracts of the supernatants of the T. reesei QM6a (blue, left *y* axis) and Δ*ypr1* (green, right *y* axis) strains, which were grown on d-glucose (liquid culture 250 ml) for 48 h. Black arrows indicate the peaks for which the absorbance spectra were measured and are provided in Fig. S1 in the supplemental material. mAU, absorbance units (in thousandths). (C) Absorbance at 370 nm of the supernatants of T. reesei QM6a (blue circles), Δ*ypr1* (green diamonds), and Re*ypr1* (yellow squares) strains, which were grown on d-glucose (liquid culture 1.5 ml) for the indicated periods. Error bars indicate the standard deviations from three independently grown cultures.

First, we sought to delete the gene that is located at the edge of the cluster in T. reesei encoding the TF 102499. For this, the uridine auxotrophic T. reesei Δ*pyr4* strain was transformed with the plasmid pCD-Δypr1, yielding a prototrophic strain lacking the coding sequence for TF 102499 (Fig. 2A). Single integration of the deletion cassette at the correct locus and successful deletion of the gene encoding TF 102499 were verified by Southern blot and PCR analyses (Fig. 2B and C). The resulting strain did not produce the typical yellow pigment on plates anymore, in contrast to QM6a (Fig. 3A). In a previous study, the yellow pigment secreted by T. reesei could be detected by reversed-phase HPLC that was actually performed to analyze the purification of a hydrophobin (4). To verify the loss of the yellow pigment synthesis in liquid culture, we extracted the yellow pigment from the culture supernatants with ethyl acetate and performed a similar HPLC analysis. In the extract of the supernatant of QM6a, several yellow compounds were detected, which were all missing in the supernatant of the deletion strain (Fig. 3B). The absorbance spectra of the crude culture supernatants and of the five main peaks detected by HPLC were determined (see Fig. S1 in the supplemental material). The supernatant of the QM6a culture had two absorption maxima (296 and 370 nm) (see Fig. S1A in the supplemental material), similar to the two main HPLC peaks at 26.1 min and 26.5 min (see Fig. S1C and D in the supplemental material). The peaks at 16.3, 30.2, and 30.9 min had only one maximum each (370 nm) (see Fig. S1B, E, and F in the supplemental material). Screening for the molecular ion of sorbicillin (*m/z* 376 of the trimethylsilyl derivative) by GC/MS revealed the presence of a strong signal in the bright yellow isolate of the major 26.5-min HPLC peak from the QM6a culture supernatant. Figure S2 in the supplemental material displays the obtained mass spectrum with the following principal ions: *m/z* (relative intensity) 376 (M^+^, 12), 361 (100), 348 (6), 335 (12), 281 (3), 207 (3), and 95 (4). All fragment ions are consistent with the molecular structure of sorbicillin: *m/z* 361 (base ion), 348, and 335 may have resulted from losses of CH_3_, CH_3_–CH, and CH_3_–CH=CH, respectively, whereas *m/z* 95 and 281 may represent the two complementary ions of a fragmentation between the aromatic ring and the side chain carbonyl. The data confirm that one of the metabolites eliminated from QM6a most likely is sorbicillin.

For a complete proof of gene function, we aimed to complement the deletion of TF 102499. To this end, the deletion strain was cotransformed with a plasmid bearing the native expression cassette of the TF 102499 (Fig. 2A, yellow frame), together with pAN7-1, a plasmid conferring hygromycin resistance (26). The presence of the expression cassette was tested by Southern blot and PCR analyses (Fig. 2B and C). The resulting complementation strain was cultivated, together with the deletion strain and their ancestor strain, on plates and in liquid culture on d-glucose. In both cases, the complementation restored the ability to produce the yellow pigment (Fig. 3A and C). Accordingly, we concluded that the TF 102499 is essential for the production of the yellow pigment(s) in T. reesei, and we termed it yellow pigment regulator 1 (Ypr1). Consequently, the deletion strain is in the following referred to as the Δ*ypr1* strain, and the complementation strain is referred to as the Re*ypr1* strain.

### Identification of the genes belonging to the cluster and the regulatory role of Ypr1.

**FIG 4:**
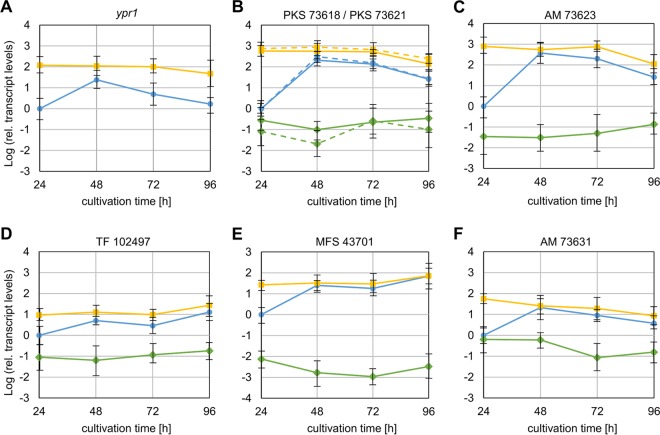
Transcript levels of the cluster genes in presence and absence of Ypr1. The T. reesei QM6a (blue circles), Δ*ypr1* (green diamonds), and Re*ypr1* (yellow squares) strains were grown on d-glucose (liquid culture, 1.5 ml) for the indicated periods. The relative transcript levels of *ypr1* (A), PKS 73618 (solid line) and PKS 73621 (dashed line) (B), AM 73623 (C), TF 102497 (D), MFS 43701 (E), and AM 73631 (F) were measured by RT-qPCR analysis, normalized by using the reference genes *sar1* and *act1*, and calculated relative to the reference sample (QM6a, 24 h). Error bars indicate the standard deviations from three independently grown cultures.

We were interested in learning to what extent Ypr1 regulates the expression of the cluster genes. To this end, we analyzed the transcript levels of all genes at this locus by RT-qPCR analysis using the mycelia of the QM6a, Δ*ypr1*, and Re*ypr1* strains grown on d-glucose (the supernatants of these samples were used for measuring the absorbance [compare Fig. 3C]). In T. reesei QM6a, the transcript levels of seven genes (indicated by arrows with black frames in the schematic overview of the cluster in Fig. 1) are upregulated after 48 h compared to 24 h (Fig. 4). This matches the time frame of the occurrence of the yellow pigment(s). Notably, the expression of two genes located in proximity to the above-mentioned seven cluster genes (encoding proteins 102492 and 102500) was not induced and is not dependent on Ypr1 (data not shown). Therefore, these genes are not considered part of the secondary metabolism cluster.

As expected, transcript levels of *ypr1* were not detected in the Δ*ypr1* strain (Fig. 4A). However, the presence of Ypr1 is essential for the expression of the two PKSs (73618 and 73621), the AM 73623, the second transcription factor (TF 10297), and the transporter (MFS 43701) since their transcript levels are barely detectable in T. reesei Δ*ypr1* (Fig. 4B to E). The expression of the AM 73631 is only upregulated in the presence of Ypr1 but does not strictly depend on it (Fig. 4F).

The expression of *ypr1* is deregulated in T. reesei Re*ypr1* (Fig. 4A). Comparing the transcript levels of *ypr1* to those of the other cluster genes, we observed a good correlation of the expression patterns both in QM6a and in Re*ypr1*. One example is the induction detected after 48 h in QM6a, whereas a constant expression was observed in Re*ypr1* (Fig. 4B to F). The only exceptions are the transcript levels of TF 102497 and the transporter 43701 at the latest time point (Fig. 4A, D, and E). Here, the transcript levels of TF 102497 and the transporter remained high, whereas the transcript level of *ypr1* was decreasing.

**FIG 5:**
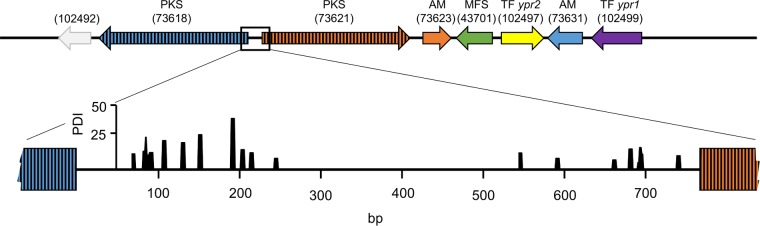
*In vivo* footprinting analyses of the bidirectional promoter between the PKS-encoding genes. The T. reesei QM6a and Δ*ypr1* strains were grown on d-glucose (liquid culture, 250 ml) for 24 h, and the DNA was methylated *in vivo* with dimethyl sulfate (DMS). Analysis of the data was performed using ivFAST (24), and the results are presented in a landscape-like visualization (25). Significant differences in methylation patterns between the two strains are given as the PDI and are plotted against the respective positions (bp) in the promoter region.

Notably, the expression patterns of the two PKS-encoding genes are nearly identical, pointing toward a bidirectional, equally strong promoter driving their gene expression (Fig. 1). To learn whether Ypr1 interacts somewhere with this region, we analyzed the whole region by *in vivo* methylation analysis comparing the T. reesei Δ*ypr1* and QM6a strains. As can be inferred from Fig. 5, the absence of Ypr1 leads to altered protein-DNA-interactions within the region, especially in proximity to the start codons of the PKS-encoding genes. These findings would be in accordance with a direct regulation expression of these genes by Ypr1.

### TF 102497 negatively regulates production of the yellow pigment(s).

**FIG 6:**
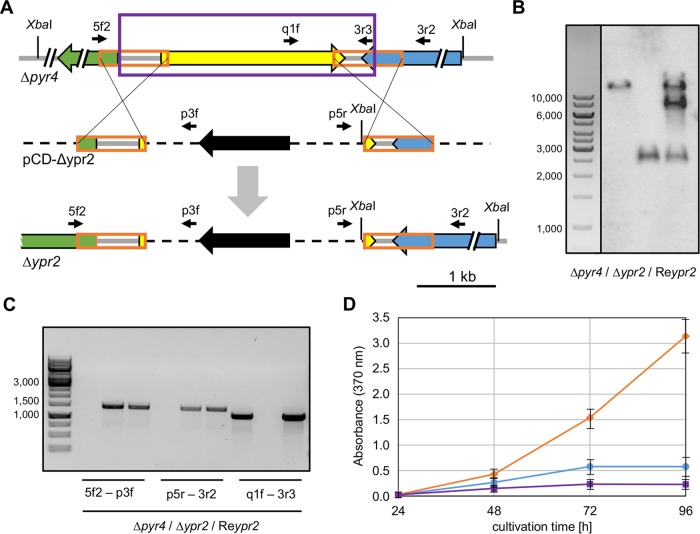
Genotypic and phenotypic characterization of T. reesei TF 102497 (Ypr2) deletion and complementation strains. (A) The uridine auxotrophic strain (Δ*pyr4*) was transformed with the plasmid pCD-Δypr2, which bears the deletion cassette, in order to replace the gene coding for the TF 102497 (yellow arrow) with the maker gene *pyr4* (black arrow) via homologous recombination, yielding a prototrophic, TF 102497 deletion strain (Δ*ypr2*). The orange frames represent 5′- and 3′-flanks for the homologous recombination. The gray arrow indicates the recombination event. Recognition sites of the endonuclease XbaI and positions of the primers (thin, black arrows) used for PCR analysis are depicted. 5f2, 102497_5fwd2; q1f, 102497_q1f; 3r3, 102497_3r3; 3r2, 102497_3r2; p3f, pyr4-3fwd; p5r, pyr4-5rev2. Gray bars indicate fungal genomic DNA, and dashed black bars indicate plasmid DNA. The purple frame indicates the expression cassette used for the TF 102497 complementation. The complementation was performed via ectopic insertion, leaving the original locus deleted. (B) Southern blot using XbaI-digested chromosomal DNA of the parent strain (Δ*pyr4*), the TF 102497 deletion strain (Δ*ypr2*), and the complemented strain (Re*ypr2*), as well as the 3′-flank as a probe. (C) Agarose gel electrophoresis of the fragments obtained by PCR with the indicated primers using chromosomal DNA of the parent strain (Δ*pyr4*), the TF 102497 deletion strain (Δ*ypr2*), and the complemented strain (Re*ypr2*) as templates. (D) Absorbance at 370 nm of the supernatants of T. reesei QM6a (blue circles), Δ*ypr2* (orange diamonds), and Re*ypr2* (purple squares) strains, which were grown on d-glucose (liquid culture, 1.5 ml) for the indicated periods. Error bars indicate the standard deviations from three independently grown cultures.

In order to determine the role of the second transcription factor in the cluster (TF 102497, Fig. 1), we deleted this gene by following a similar strategy as that used for the deletion of Ypr1. By homologous recombination, the gene coding for TF 10297 was replaced with a *pyr4* marker cassette (Fig. 6A). To this end, the T. reesei Δ*pyr4* strain was transformed with pCD-Δypr2. Single integration of the deletion cassette at the correct locus and successful deletion of the gene encoding for TF 102497 were verified by Southern blotting and PCR analyses (Fig. 6B and C). Next, we constructed a complementation strain that bears the native expression cassette of TF 102497 (Fig. 6A, purple frame) ectopically inserted into the chromosome of the deletion strain via cotransformation with pAN7-1 (26). The presence of the expression cassette was tested by Southern blotting and PCR analyses (Fig. 6B and C). For an initial phenotypic characterization, we cultivated the resulting complementation strain, the deletion strain, and their ancestor strain in liquid culture on d-glucose. We detected a larger amount of yellow pigment(s) in the TF 102497 deletion strain than in QM6a (Fig. 6D). The complementation was able to restore the QM6a phenotype (Fig. 6D). Based on its regulatory influence on the synthesis of the yellow pigment(s), we termed the TF 102497 yellow pigment regulator 2 (Ypr2). Consequently, the deletion strain is referred to as the Δ*ypr2* strain and the complementation strain is referred to as the Re*ypr2* strain below.

### Ypr2 represses gene expression of most genes of the cluster, including *ypr1*.

**FIG 7:**
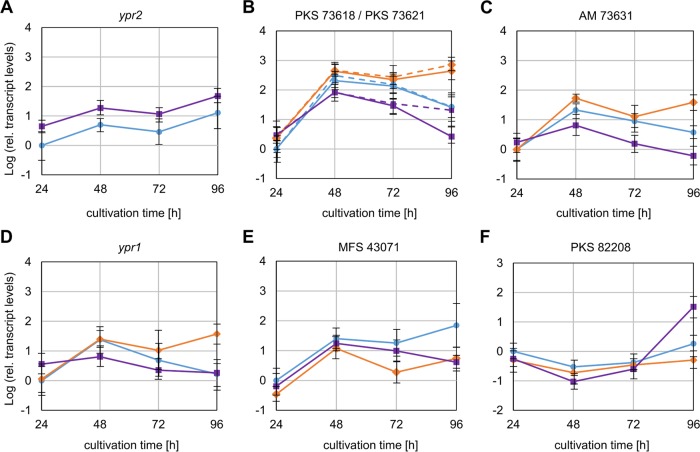
Transcript levels of the cluster genes in presence or absence of Ypr2. The T. reesei QM6a (blue circles), Δ*ypr2* (orange diamonds), and Re*ypr2* (purple squares) strains were grown on d-glucose (liquid culture, 1.5 ml) for the indicated periods. The relative transcript levels of *ypr2* (A), PKS 73618 (solid line) and PKS 73621 (dashed line) (B), AM 73631 (C), *ypr1* (D), MFS 43701 (E), and PKS 82208 (F) were measured by RT-qPCR analysis, normalized by using the reference genes *sar1* and *act1*, and calculated relative to the reference sample (QM6a, 24 h). Error bars indicate the standard deviations from three independently grown cultures.

In order to gain more detailed insights into the regulatory impact of Ypr2, we investigated the transcript levels of the cluster genes in the absence or presence of Ypr2. Mycelia of the QM6a, Δ*ypr1*, and Re*ypr1* strains grown on d-glucose were tested by RT-qPCR analysis (the supernatants of these samples were used to measure the absorbance [compare Fig. 6D]). As expected, the transcript levels of *ypr2* could not be detected in Δ*ypr2*; in the Re*ypr2* strain they were four times higher than in QM6a (Fig. 7A). The transcript levels of the PKS-encoding genes (73618 and 73621) (Fig. 7B) and the auxiliary modifiers, 73631 (Fig. 7C) and 73623 (data not shown because redundant), were elevated or similar in the Δ*ypr2* strain and lower in T. reesei Re*ypr2* compared to QM6a. Their expression patterns are similar compared to the pattern of *ypr1* (Fig. 7D). We therefore suppose that Ypr2 regulates the expression of Ypr1 and thereby indirectly influences the expression of other cluster genes. The regulatory influence of Ypr2 is most pronounced at late time points (96 h). Then, the transcript levels of *ypr1*, the PKS-encoding genes, and the auxiliary modifiers are regulated in the presence of Ypr2, but they remain at high levels in the Δ*ypr2* strain (Fig. 7B to D). Notably, the transcript levels of the transporter 43701 do not fit into this picture (Fig. 7E). As in the case of all other cluster genes, its transcription is induced after 48 h (Fig. 7B to E). In contrast to the other cluster genes, its transcript level is lower in the absence of Ypr2 than in the presence of Ypr2 (Fig. 7E, 72 h). This suggests that the gene expression of the transporter 43701 is positively regulated by both Ypr1 and Ypr2. Ypr1 is essential for gene expression and the induction, whereas Ypr2 is necessary for keeping the transcript levels high. It needs to be mentioned that in Chaetomium globosum the gene encoding a transcription factor that is most similar to *ypr2* (TF 08141) is located outside the cluster on another scaffold (compare Fig. 1). There, it is positioned directly in front of a PKS-encoding gene (PKS 08141); in fact, the gene model fuses the two genes. Interestingly, this PKS has a homolog in T. reesei, namely, protein ID 82208. To test whether Ypr2 might regulate its expression, we measured the transcript levels of this gene. We were unable to observe an influence of Ypr2 on the expression of the PKS 82208 in T. reesei at early time points (Fig. 7F). However, the high transcript level in T. reesei Re*ypr2* after 96 h points to a regulation of PKS 82208 expression by Ypr2.

## DISCUSSION

During this study, we found that the typical yellow pigment secreted by T. reesei is a mixture of several yellow compounds, as indicated by HPLC data. In contrast, Nakari-Setala et al. detected only a single yellow compound (4). One possible explanation is that Nakari-Setala et al. aimed at purifying a hydrophobin and performed either freeze-thaw cycles or foaming by injecting air, whereas in the present study the supernatant was extracted with ethyl acetate. Besides this, the presence of several yellow compounds may be explained by the presence of different naturally produced yellow pigments. However, it may also be the result of degradation and modifications of a single yellow pigment after biosynthesis during cultivation and/or sample processing.

**FIG 8:**
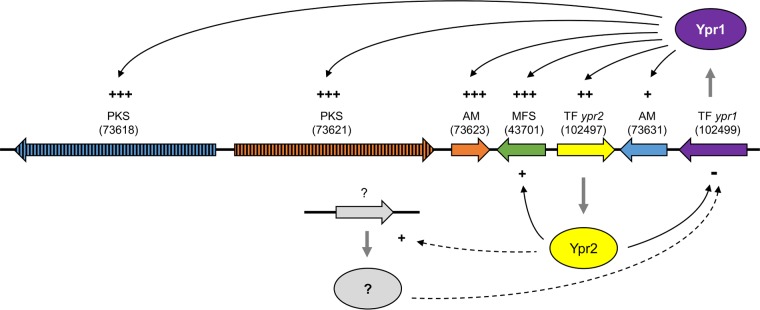
Schematic drawing of the regulatory model involving the two transcription factors Ypr1 and Ypr2. Transcription factors are depicted as ovals, genes are depicted as black-rimmed arrows, regulatory influences demonstrated in this study are depicted as thin black solid arrows, and alternative regulatory routes are depicted as thin black dashed arrows. Plus or minus symbols indicate a positive or a negative regulatory influence, respectively, and the number of operators indicates the extent. Question marks indicate unknown, putatively involved regulators and their encoding genes. PKS, polyketide synthase; AM, auxiliary modifier; MFS, transporter of the multifacilitator superfamily; TF, transcription factor. Protein IDs are given in parentheses.

The deletion of the main activator, Ypr1, resulted in the loss of the ability to produce the yellow pigment(s) and the near abolishment of the transcription of the entire cluster genes. The transcript levels of the cluster genes match the amounts of synthesized yellow pigment(s) in all investigated strains. Taken together, this strongly supports that this cluster is responsible for the synthesis of the yellow pigment(s). It is generally acknowledged that transcription factors of secondary metabolism clusters act as narrow-range regulators; however, it cannot be excluded that Ypr1 and/or Ypr2 might regulate the expression of genes outside the cluster. We found that the presence of Ypr2 represses the expression of most genes of the investigated cluster, including *ypr1*. However, Yrp2 is a Gal4-like transcription factor and, as such, one would expect a transactivating function. An explanation would be that Ypr2 activates the expression of a repressor, which in return regulates the transcription of *ypr1*. This model would match the observed delay between first induction of *ypr2* (48 h) and the downregulation of *ypr1* expression (later than 48 h) (Fig. 7A and D). We have visualized the proposed regulatory model and the involvement of both transcription factors (Ypr1 and Ypr2) in Fig. 8. However, given that (i) the gene coding for the transporter 43701 and *ypr2* are oriented in a tail-to-tail manner and share a 544-bp intergenic region as the putative regulatory region for both genes (Fig. 1), (ii) the transcript levels of both genes remain high (Fig. 7A and E), although the transcript level of the activator *ypr1* is decreasing (Fig. 7D), and (iii) Ypr2 contributes to keep the expression of the transporter (43071) at a high level, we speculate that Ypr2 might positively act on its own gene expression.

Although the expression of all other investigated genes from the cluster require the presence of Ypr1, the expression of the AM 73631 is induced by Ypr1 but does not depend on it. The fact that it is expressed at a considerable level also in the absence of Ypr1 suggests that it is involved in metabolic processes other than the synthesis of the yellow pigment(s). This idea is supported by the finding that the gene encoding its homolog in U. virens (UV8b_1551) is located outside the cluster on another scaffold. In P. chrysogenum, Chaetomium globosum, and both Colletotrichum species, no homologs of T. reesei AM 73631 or A. chrysogenum ACRE_048110 could be identified. However, these organisms—and many other ascomycetes—possess proteins with notable sequence similarities to A. chrysogenum ACRE_048110 (with ca. 40% identity). It appears as though the auxiliary modifier ACRE_048110/AM 73631 might be involved in other metabolic process and that its activity in the biosynthesis of the yellow pigment(s) is either not essential or can be substituted by the activity of similar proteins. In P. chrysogenum, the auxiliary modifier Pc21g05110 was suggested to be part of the cluster based on expression analyses (15). However, its homologs or the most similar proteins in the other organisms containing the cluster are located somewhere else in their respective chromosomes. In U. virens and Chaetomium globosum other auxiliary modifiers seem to be part of the cluster (Fig. 1). On the other hand, some auxiliary modifiers are missing in the cluster in the different organisms. As mentioned before, homologs of the A. chrysogenum ACRE_048110 and the T. reesei 73631 are absent in P. chrysogenum and also in Chaetomium globosum, Colletotrichum graminicola, and Colletotrichum sublineola. Further, homologs of A. chrysogenum ACRE_048140 are only found in Colletotrichum graminicola and Colletotrichum sublineola. Summed up, it seems as though the organisms have modified the auxiliary biosynthesis machinery around the core PKS mechanism in order to produce different compounds.

## Supplementary Material

Supplemental material
