# Supplementary material for: Identification of the Main Regulator Responsible for Synthesis of the Typical Yellow Pigment Produced by Trichoderma reesei
Source: Appl Environ Microbiol. 2016 Sep 30;82(20):6247–57. doi: 10.1128/AEM.01408-16 (PMC5068150; doi:10.1128/AEM.01408-16)
Supplement: Supplemental material [file supp_82_20_6247__index.html]

Supplemental material 

# Identification of the Main Regulator Responsible for Synthesis of the Typical Yellow Pigment Produced by Trichoderma reesei

## Supplemental material

- Supplemental file 1 -

  Absorbance spectra of supernatants and five main peaks from HPLC analysis (Fig. S1) and mass spectrum obtained by GC/MS analysis of the major yellow metabolite isolated from the supernatant of QM6a by preparative HPLC (Fig. S2).

  PDF, 519K
