# Supplementary material for: Identification of the Main Regulator Responsible for Synthesis of the Typical Yellow Pigment Produced by Trichoderma reesei
Source: Appl Environ Microbiol. 2016 Sep 30;82(20):6247–57. doi: 10.1128/AEM.01408-16 (PMC5068150; doi:10.1128/AEM.01408-16)
Supplement: Supplemental material [file AEM.01408-16_zam999117467so1.pdf]

Fig. S1

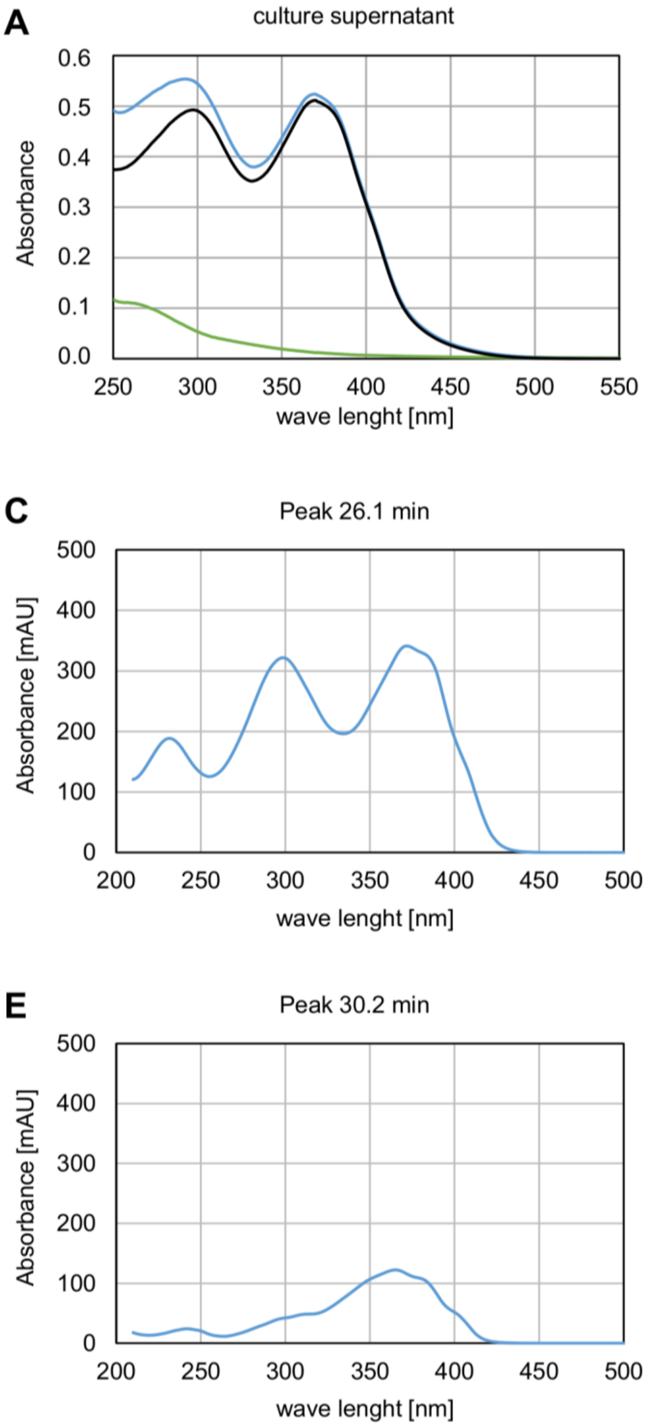

FIG S1 Absorbance spectra of supernatants and five main peaks from HPLC analysis. The *T. reesei* strains QM6a (blue) and Δ*ypr1* (green) were grown on D-glucose (liquid culture 250 ml) for 48 hours. The absorbance of 1:8 dilutions (in ddH_2O) of the respective culture supernatants were measured. Subtraction of the two spectra yields the spectrum of the yellow pigment(s) (black). The absorbance of the five main peaks obtained by reversed-phase HPLC of the ethyl acetate extracts of the supernatant of QM6a was measured.

Fig. S2

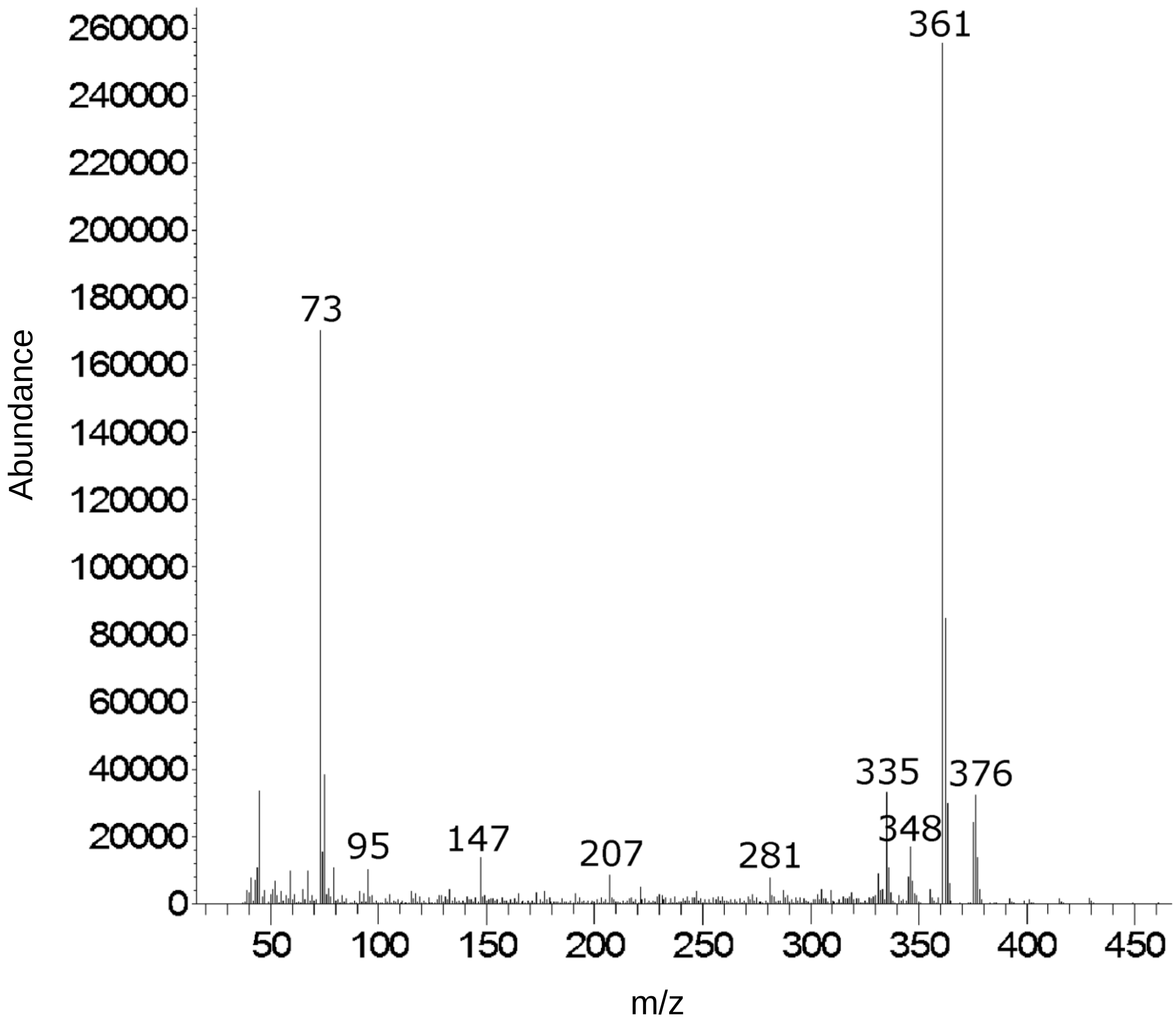

FIG S2 Mass spectrum obtained by GC/MS analysis of the major yellow metabolite (26.5 min HPLC peak in Fig. 3B and Fig. S1D) isolated from the supernatant of QM6a by preparative HPLC. m/z values of the most abundant ions are indicated, of which 376, 361, 348, 335, 281 and 95 may be diagnostic for TMS sorbicillin; 73 and 147 are from silyl ions.
